# Exposure of Prostate to Lipopolysaccharide and Hypoxia Potentiates Neoplastic Behavior and Risk for Prostate Carcinogenesis In Vivo

**DOI:** 10.1155/2014/420429

**Published:** 2014-08-17

**Authors:** Maxwell Omabe, Kenneth Omabe, Martin Okwuegbu, Ogo Grace, Desmond Uchenna Okoro

**Affiliations:** ^1^Department of Oncology, Cancer Research Division, University of Saskatchewan, Saskatoon, SK, Canada S7N 5R5; ^2^Molecular Cancer Biology Research Group, Molecular Pathology and Immunology Division, Department of Medical Laboratory Sciences, School of Biomedical Science, Faculty of Health Science, Ebonyi State University, Abakaliki, Nigeria; ^3^Department of Pathology and Molecular Medicine, University of Leicester, Leicester, UK

## Abstract

A number of studies showed that men from tropical countries have higher burden of prostate cancer similar to data from USA. We developed a translational model to examine whether exposure to microbial inflammation-inducing molecule lipopolysacchride LPS was associated with prostatic cell transformation to more proliferative phenotype as indicated by PSA secretion. Immunocompetent adult mice were divided into two groups; the first group received a local prostate inoculation with *E. coli*, while the second group received inoculation with sterile solution of saline as vehicle. At the end of 6 days, the PSA values were measured and compared. In the second experiment, two groups of animals were involved. The test group received two drops of the hydrogen peroxide orally for six to seven days to induce hypoxia, while the control group received normal saline. Blood samples were evaluated for serum level of PSA. Result showed a 2-fold increase in level of PSA compared to the control mice in the *E. coli* inoculated-LPS exposed animals. In addition, exposure of the animals to hypoxic stress resulted in 3.5 fold increase in the serum PSA compared to the control group, which was found to be statistically significant (*P* < 0.0001). In conclusion, our data shows that chronic prostatic infection and exposure to inflammatory stimulus, especially LPS, may alter the phenotype of prostate epithelial cells for increased PSA secretion, a known cancer-like behavior; this is mediated by compromised redox state and oxidative stress injury. We propose that exposure of the prostate epithelial cells to lipopolysaccharide (LPS) promotes chronic inflammation and risk of neoplastic behavior of the prostate in vivo; this may explain the high rate of prostate cancer in tropics.

## 1. Introduction

Prostate cancer is a form of cancer that develops in the male reproductive system often as slow growing mass before it is transformed to a malignant state; at this stage, the cancerous growth and tumor mass may become more aggressive [1, 2]. The cancer cells may metastasize from the prostate to other parts of the body, particularly the bone and lymph nodes [3, 4]. Globally, prostate cancer is the sixth leading cause of cancer-related death in men [2]. In the United States, it is the second leading form of cancer in men [5]. Rates of detection of prostate cancers vary widely across the world, with South and East Asia detecting less frequently than in Europe and especially in America [1]. Recent studies have shown that Africa American men have the highest mortality rate of prostate cancer compared to any other racial or ethnic group in [6].

The population-based studies on prostate cancer disparity were based on African American men only or compared African American men to other racial groups within the USA. With the problem of prostate cancer disparity persisting, Odedina et al., in 2009, examined the problem of prostate cancer disparity from a new perspective, the findings of their studies have allowed us to develop a new paradigm regarding the growing literature on the disproportionate burden of prostate cancer among other black men within West African subregion, including Nigeria and Ghana [6]. The study clearly showed higher rate of prostate cancer in West Africa. Clearly, the authors' report opens a new perspective for investigation into problems of prostate cancer burden within the sub-Saharan Africa, from the angle of the cause or the source, and moving away from the most traditional reports of genetic basis of prostate carcinogenesis [1, 7] to a possibility of inflammation-mediated prostate carcinogenesis [8, 9]. In addition, abundant evidence suggests a potential and important influence of environmental factors acting on prostate cancer risk. Complete understanding of the causes of prostate cancer remains elusive [8, 10, 11]. We have shown that inflammation of the prostate might increase the risk of developing prostate cancer [8, 9]. Importantly, data from the Laboratory of the authors clearly suggest that lack of access to antibiotics and presence of chronic inflammatory molecules might be associated with prostate cancer risk. We propose that increasing incidence of prostate cancer among West African men [6] might be due to constant exposure to microbial infection, which might induce inflammation-mediated hypoxia-driven cancer-like behaviors in the prostate. To test this, an experimental model was developed to investigate if exposure to microbial inflammatory molecule is linked to alteration in prostatic epithelia cell phenotype and cancer like behavior. Here we hypothesize that exposure of the prostatic epithelial tissue to lipopolysaccharide from gram negative bacteria and hypoxic stress may induce prostate cancer associated cellular behaviors in mice, with important implication in human. We show that chronic local exposure prostate to lipopolysaccharide and superoxide resulted in increased PSA secretion, indicating a trend to prostate cancer transformation.

## 2. Material and Method 

### 2.1. Animal Experiment

A total of 30 adult male mice weighing between 130 and 220 g were used for these study; the animals were obtained from animal house at Enugu State University, department of animal science. On arrival, the animals were placed at random and allocated for treatment in a wire gauze cage with paddy husk as bedding. Animals were housed at a temperature of 24 ± 3°C in Ebonyi State University research laboratory where all the animals were allowed to free access to fed and water (with standard commercial polluted rat chaw) without any treatment for 2 weeks, and this is to allow them to acclimatize. At the end of the 2 weeks, the animals were divided into 2 groups. Group-1 was 5 in number and served as the control group without treatment whereas group-2 was 10 in number and serves as the test group.

### 2.2. Experimental Prostatitis with Lipopolysaccharide

The weight of the animals was measured for both test and control. A pure colony of* E. coli* measuring 1 × 10^5^ (obtained from federal teaching hospital Abakaliki) was emulsified on 10 mL of normal saline on a plain container and was allowed to completely dissolve. A 2 mL syringe was used to inject directly into the prostate of the mice 0.2 mL of the bacteria solution. The animals were allowed to access freely food and water both test and control. The control animals were treated same as the test but with normal saline only for control group. The animals were closely observed on daily bases and were carefully monitored for at least 6 days. The animals' weights were measured and recorded. All animals were handled as specified by ethical committee for animal right. 2 mL of blood was collected from each mouse by cardiac puncture for both the test and control into a plain (anticoagulant free) container and allowed to clot. Then those samples were centrifuged at 3000 g per minute at room temperature. The serum was separated and stored in the freezer at −20°C at Ebonyi State University Research Laboratory.

### 2.3. Treatment with Hydrogen Peroxide

Group-A which was five in number served as the control group. Group-B was ten in number and served as the test group. All members of group B received two drops of hydrogen peroxide orally twice daily, while their weights were also measured daily. The two groups were monitored for at least 6 days. The animals' weights were measured and noted. All animals were handled as specified by ethical committee for animal right. 2 mL of blood was collected from each mouse by cardiac puncture for both the test and control into a plain (anticoagulant free) container and allowed to clot. Then those samples were centrifuged at 3000 g per minute at room temperature. The serum was separated and stored in the refrigerator at −20°C at Ebonyi State University Research Laboratory.

### 2.4. Laboratory Analysis

Prior to the time of analysis, the samples were allowed to return to room temperature by removing the samples from the refrigerator. Each serum sample from the two separate experiments was analyzed for “prostate specific antigen” (PSA) using enzyme immunoassay with microtitre wells. The principle of the Elisa test was based on solid phase enzyme linked immunosorbent assay technique. The assay system utilizes a goat anti-PSA antibody directed against PSA for solid phase immobilization (using the microtiter plate). A monoclonal anti-PSA antibody directed against PSA antigen. A monoclonal anti-PSA antibody conjugated to horseradish peroxidase (HRP) was in the antibody-enzyme conjugate solution. The test was performed according to the manufacturer's instruction. The test samples were allowed to react first with the immobilized goat antibody at room temperature for 60 minutes. The wells were washed to remove any unbounded antigen. The monoclonal anti-PSA-HRP conjugate was reacted with the immobilized antigen for 60 minutes at room temperature, resulting in the PSA molecules being sandwiched between the solid phase and enzyme linked antibodies. The wells were washed with water to remove unbound labeled antibodies. A solution of TMB reagent was added and incubated at room temperature for 20 minutes, resulting in development of a blue color. Color development is stopped with the addition of stop solution changing the color to yellow. The concentration of PSA is directly proportional to the color intensity of the test sample. Absorbance is measured spectrophotometrically at 450 nm wavelength.

## 3. Result

### 3.1. Experiments with* E. coli* and Lipopolysaccharide (LPS) to the Prostate

**Figure 1:**
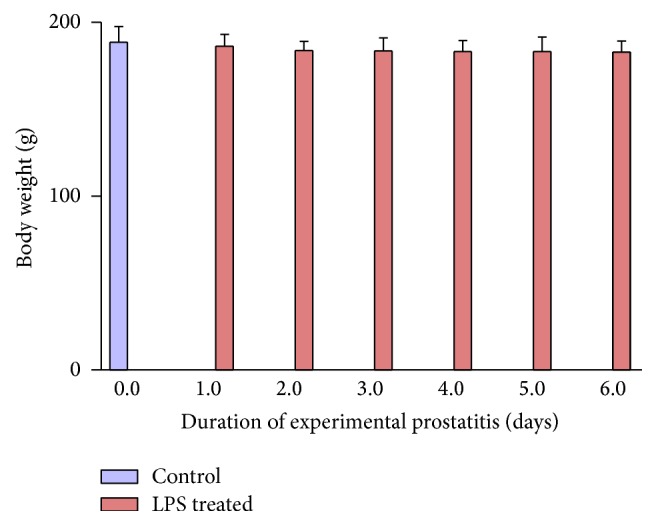
Shows variation in weight of the mice with local exposure of the prostate to lipopolysaccharide from* E. coli*, in comparison with the untreated group (blue bar). This experiment was repeated at least 3 times. Clearly, there was no statistical significant difference in their mean weight from day 2 of the experiment which was maintained to 6 days later.

The data from all the groups were recorded and analyzed with Prism statistical software. From Figure 1 shows the relationship in weight between the control group before the animals were exposed to local prostatic infection and prostatitis and the weight of the mice during the days of the experimental prostatitis. As shown below, the weight of the test mice was gradually decreasing on daily basis; this was suspected to be due to increase in inflammatory response of which lipopolysaccharide (LPS) is generally known for, by generating substantial level of cytokine. Accordingly, it was observed that the animals developed poor appetite. This was expected since chronic infection is a known cause of poor appetite. In addition, there was mild a reduction in weight (Figure 1) which was not statistically significant in the animals whose prostate was exposed to LPS for compared with the control group. This might be due to the short duration of the experiment, perhaps longer duration or the chronic inflammatory state could result in more significant changes in weight as many other parameters would also be affected.

**Figure 2:**
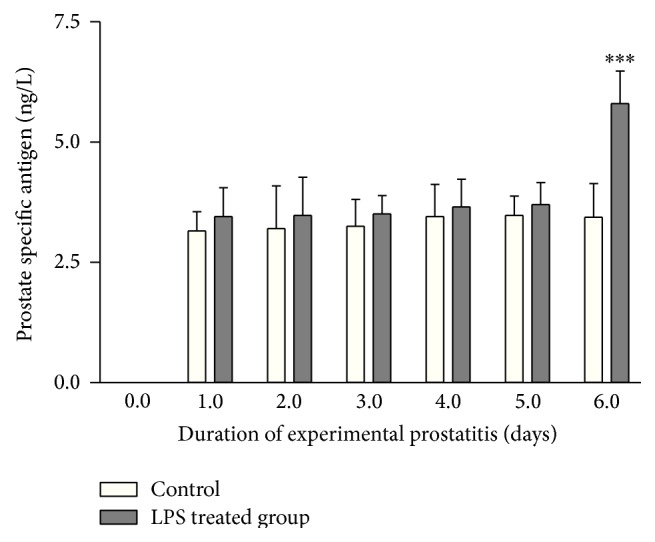
Illustrates the gradual increase in serum PSA levels in experimentally induced prostate infection in immunocompetent mice when compared to the control group. The animal had their dorsal prostate local exposed to 1 × 10^5^ concentration of* E. coli* as the source of lipopolysaccharide. The animals were allowed in a conducive environment throughout the period of the study. PSA level was determined as described in the materials and method. Data were compared with the untreated group. The experiment was repeated 3 times and the SD determined. *n* = 3. Error bar represent standard deviation.  ^***^ = *P* < 0.0001.

Figure 2 shows the PSA level of test mice before and after exposure of the animal's prostate to LPS and development of experimental prostatitis compared to the control group. It was found that from day 1 of the experiment, there was a gradual increase in serum PSA when compared to the control; on day 6 of the experiment, a very high level of PSA was observed, which showed statistically significant increase (*P* < 0.0001), suggesting that infection of the prostate epithelium and exposure to LPS may mediate state of local epithelial chronic inflammatory condition that may interfere with prostatic epithelial cell phenotype and result in prostate-cancer-like behavior in vivo in murine model.

### 3.2. Inducement of Hypoxic Stress with Hydrogen Peroxide

To understand why exposure to LPS would result in significant increase in PSA secretion; since LPS is a known stimulant for inflammation, we proposed that infiltrating inflammatory molecules and change in the redox state of prostate microenvironment might induce a hypoxia similar to that found in solid tumor and could compromise the genomic stability of the epithelia cells, resulting in programming of the cells to a phenotype with cancer-like behaviors, including increase in PSA secretion. Published studies have shown that hypoxia selects cells with more malignant phenotype and reprograms the genome of cancer cells for more aggressive behavior [12–14]. To investigate this, a prostate microenvironment that mimics the effect of hypoxic stress was induced according to established protocol and published [15]. This was done using adult male mice. Ten out of the fifteen adult male mice used for the study were successfully induced (fed orally) with hydrogen peroxide (two drops twice daily) for seven days, after which the prostate specific antigen (PSA) value was measured as described in the material and method. Result showed a statistically significant increase in serum PSA (*P* value <0.0025) at 95% confidence limit when compared with the control group. Of particular note was that the PSA level increased 3.5-fold compared to the control group.

## 4. Discussion 

Prostate cancer has been shown to be the second leading cause of cancer-related death in men hence accounting for 37% of all cancer cases globally [5]. Available evidence suggests that the burden of prostate cancer in West African including Nigeria and Ghana is very high [16]. For example, Wiredu and Armah in 2006 [17], conducted a 10-year review (1991–2000) of all cancer mortality patterns at the same institution based on autopsy and death certification data and found prostate cancer to be the second leading cause of cancer deaths (286 cases). Contrary to the WHO IARC global ranking, several published studies indicate higher incidence of prostate cancer in Nigerian men [16]. In fact the authors provided documented evidence in the literature indicating that prostate cancer is high in West African; Nigeria and Ghana had a similar rate to what is found in the United States and in Caribbean Islands.

Whereas the genetic contribution to carcinogenesis is generally established, emerging evidence from both the authors Laboratory and others strongly suggest an inflammatory role for prostate carcinogenesis in animal and clinical studies [8–10, 18, 19]. These studies have provided convincing evidence that suggest that apart from genetic factors, environmental factors including infection and inflammation may be a leading cause of prostate carcinogenesis, especially in the tropical countries. To examine the cause of high prostate cancer trend in Nigeria and Ghana, we developed preclinical models of experimental prostatitis and hypoxia in animal model and proposed that chronic exposure to microbial inflammatory molecules including LPS may result in inflammation-mediated hypoxia-driven prostate carcinogenesis. Data have shown that exposure of the prostate to LPS from gram negative bacteria was associated with statistically significant decrease in weight and increase in PSA secretion (Figures 1 and 2). The result of the current studies confirms our previous report [8] and supports the reports of de Marzo et al. 1999 [20] and that of Sfanos and de Marzo 2012 [21].

The current study provides data which points to an important role of chronic prostatic infection especially due to gram negative bacteria or implication of exposure to LPS in prostate epithelia cell proliferation, indicated by the level of PSA in animal models. We have shown that exposure of LPS to the prostate epithelial cells in murine model resulted in inflammation-mediated significant increase in PSA secretion compare to the control group treated with normal saline. Our results are consistent with the findings of Masnaon et al., 1999 [22]; in that study, the authors measured the PSA of 6 patients diagnosed with chronic prostatitis and observed that their PSA level was all elevated; however, after an antibiotic treatment all except 1 patient had their PSA reversed to a normal serum levels. In that study, a histopathological examination of formalin fixed paraffin embedded sections of the prostate samples from the patient's demonstrated evidence suggestive of presence of adenocarcinoma. Furthermore, injury caused by pathogens or proinflammatory cytotoxic agents have been shown to trigger proliferation of prostatic glandular cells, leading to appearance of epithelial lesions known as “proliferative inflammatory atrophy” (PIA), in a recent report of Vral et al., 2012 [23]; The author showed that inflammatory cells infiltrating the prostate released genotoxic reactive oxygen species, leading atrophic cells to neoplastic progression.

That study also examined the link between PIA or prostatic intraepithelial neoplasia (PIN) and to inflammation or clinical prostatitis, by analyzing 1367 prostate biopsies from 98 patients with a recent history of chronic prostatitis, and 32 patients with biopsies positive for carcinoma, and demonstrated that PIA was found more frequently in biopsy cores containing a severe or moderate inflammatory focus, compared to non-PIA lesions [23]. The authors clearly indicated that the extent of PIA per patient correlated with the burden of moderate or severe inflammation; and that low-grade PIN was found in over 90% of cases emerging from normal, nonatrophic glands which were more frequently found in biopsy cores with absent or mild inflammatory burden [23]. Data from that study provide support to our current findings in a clinical perspective and point to a positive association between tissue infection, inflammation, clinical prostatitis, and the putative cancer risk-lesion PIA. The increase in PSA was an indication of proliferative state of the prostate [24]; it is generally accepted that chronic inflammation often triggers highly proliferative episodes, especially in the setting of longstanding or prolonged state of inflammation. Carcinoma of the liver, large bowel, urinary bladder, and gastric mucosa has been reported [25]. Our data have shown a risk of prostate cancer in the setting of chronic inflammation induced by local exposure to LPS in experimental model, as in Figure 2, which agrees with both experimental and clinical studies.

**Figure 3:**
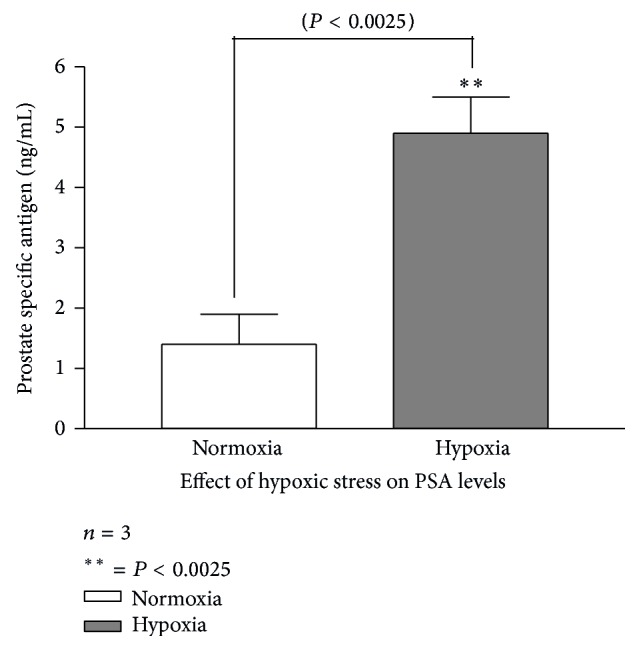
Adult male mice were experimentally induced to hypoxic stress by daily treatment with hydrogen peroxide and the control group received only normal saline. Blood samples were collected from each group after 6 days and were assayed for serum PSA by ELISA method. Data showed that exposure of the prostate to hypoxic condition resulted in 3.5-fold increase in serum PSA level compared to the control, demonstrating a statistical significance (*P* < 0.0025). Data represents mean and error bar = SEM (*n* = 3).

**Figure 4:**
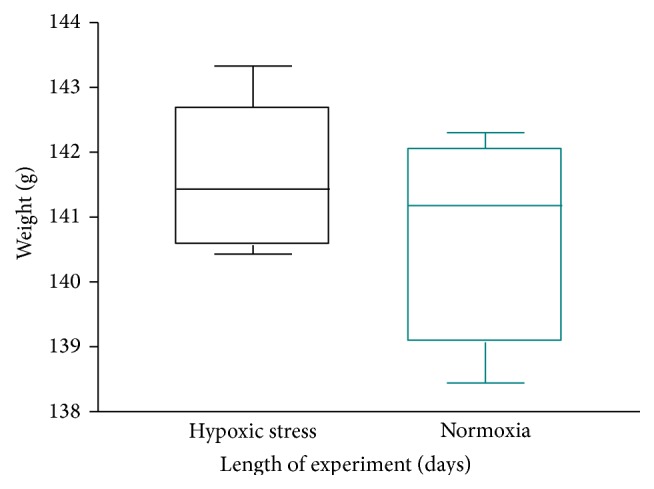
Represents the mean weight of animals treated with hydrogen peroxide 2 drops twice daily for at least 6 days and the controls that received normal saline 2 drops twice daily. Data show that exposure to hypoxia and oxidative stress did result in significant change in the weight of hydrogen peroxide treated group in comparison to the control group.

To understand the mechanism for the observed LPS induced inflammation mediated cancer risk, the effect of hypoxia and oxidative stress on the epithelial cells of the prostrate was investigated. Much evidence has indicated that hypoxia (both chronic and acute/cyclic) affects various aspects of tumorigenesis frequently through induction of the hypoxia-inducible factor (HIF) transcription factors [26]. Data showed that systemic and local state of hypoxia resulted in a 3.5-fold increase in PSA level (Figure 3) compared to animals in normoxic state, with no observable change in the weight of both test and control group (Figure 4). Indeed, animals that received daily dose of hydrogen peroxide had statistically significant increase in PSA secretion compared to the control group that received saline as vehicle. The result was consistent with the report of Butterworth et al., 2008 [12], which showed that hypoxic prostate epithelia cell model, LNCaP-H1 subline, showed altered growth characteristics and exhibited androgen independent growth both in vitro and in vivo. Growing evidence from experimental and clinical studies points to the fundamental, pathophysiologic role of inflammation and hypoxia in carcinogenesis [3, 27]. It appears that intratumoral hypoxia may be a consequence of a structurally and functionally disturbed microcirculation which is known to occur during inflammation [4]; by deteriorating oxygen diffusion geometry in the affected organ or tissue, and a consequence alteration in gene expression and proteome that can activate stress response and promote tumor initiation and propagation [4, 12, 13, 26]. Put together, data from the current study suggests that the observed phenomenon with LPS and prostatitis may be driven by hypoxia associated prostate inflammation. It appears that hypoxia following sustained inflammation may reprogram cells lining the acini and ducts of prostate gland for increased cell proliferation, which allows for cumulative increase in the total secretion of PSA. It is generally accepted that increase in the serum level of PSA, a well-recognized diagnostic marker for prostate cancer and its progression, is an early marker for neoplastic behavior.

In conclusion, we propose that exposure of the prostate to LPS may induce local chronic inflammation; this results in generation of local oxidative damage that promotes neoplastic behavior in prostatic epithelial cells. This may in part explain the high prostate cancer burden in West Africa, including Nigeria and Ghana, as prostatitis due to the fact that bacterial infection is quite common.
